# The Treatment of Neuralgia

**Published:** 1880-06

**Authors:** 


### ARTICLE XI.

### *The Treatment of Neuralgia.*

Aconite is an old remedy in Neuralgia which has, however, not altogether realized the expectations which were formed of its value. The power which it often lacks, has been lately claimed for its alkaloid by Professor Gubler, who announced that aconitia is almost infallible in trigeminal neuralgia. This substance was long banished from the materia medica for internal use, but it has been employed occasionally since the discovery of a crystallized form by Gréhaul and Duquesnel in 1871. Its value in neuralgia has lately been investigated by the New York committee on Neurotics, of which Dr. E. C. Sequin is the chairman. The dose of all forms of aconitia is about the same. The initial dose being about half a milligramme (or 1-130 grain) twice or thrice a day. There are differences in susceptibility and some persons cannot bear a larger dose than 1-200 grain; while one case was met in which 1-84 grain every three hours was tolerated. From a trial of the treatment in a series of cases, the committee conclude that, on the average, distinct physiological and therapeutical effects may be obtained by giving 1-100 grain three times a day. Of six cases of severe trigeminal neuralgia, one, probably a reflex neuralgia from a decayed tooth, was not at all benefited. Three cases of epileptiform neuralgia were slightly, or only temporarily relieved. Two cases were cured. One of them had existed for seven years, with an interruption of seven months, procured by resection of the affected nerve. The results thus afford a partial support to M. Gubler's assertion.

The value of ammoniacal sulphate of copper in the treatment of the same affection has been asserted by Mr. Fereol in a recent communication to the Academie de Medicine He states that in cases in which every treatment has failed even the administration of gelseminum and of aconitia, a cure or remarkable relief may be obtained to the most severe symptoms by this drug.

Among the examples he gave of its use was the following: Trifacial of two months' duration, with absolute (?) insomnia, was unrelieved by the extraction of teeth, quinine, bromide, aconitia, or tincture gelseminum, hypodermic injections of morphia or arsenic. From the first day of the administration of the ammonia sulphate of copper, there was a notable remission in the symptoms and cessation of the insomnia. In one case, the dose was pushed to eight grains, without any other accident than nausea. It has the drawback of occasioning a persistent metallic taste in the mouth. One case of intolerance was met with; in that a grain and one-half of sulphate of copper occasioned violent vomiting.—*London Lancet.*

